# Pharmacy students can improve access to quality medicines information by editing Wikipedia articles

**DOI:** 10.1186/s12909-018-1375-z

**Published:** 2018-11-20

**Authors:** Dorie E. Apollonio, Keren Broyde, Amin Azzam, Michael De Guia, James Heilman, Tina Brock

**Affiliations:** 1 Department of Clinical Pharmacy, University of California, San Francisco, San Francisco, California USA; 2 King’s College London, London, UK; 3 Department of Psychiatry, University of California, San Francisco, San Francisco, California USA; 4 Kaiser Permanente, Panorama City, California USA; 5 Department of Emergency Medicine, University of British Columbia, Vancouver, BC Canada; 6 Faculty of Pharmacy and Pharmaceutical Sciences, Monash University, Melbourne, Australia

**Keywords:** Students, pharmacy, Pharmacy, Curriculum, Wikipedia

## Abstract

**Background:**

Pharmacy training programs commonly ask students to develop or edit drug monographs that summarize key information about new medicines as an academic exercise. We sought to expand on this traditional approach by having students improve actual medicines information pages posted on Wikipedia.

**Methods:**

We placed students (*n* = 119) in a required core pharmacy course into groups of four and assigned each group a specific medicines page on Wikipedia to edit. Assigned pages had high hit rates, suggesting that the topics were of interest to the wider public, but were of low quality, suggesting that the topics would benefit from improvement efforts. We provided course trainings about editing Wikipedia. We evaluated the assignment by surveying student knowledge and attitudes and reviewing the edits on Wikipedia.

**Results:**

Completing the course trainings increased student knowledge of Wikipedia editing practices. At the end of the assignment, students had a more nuanced understanding of Wikipedia as a resource. Student edits improved substantially the quality of the articles edited, their edits were retained for at least 30 days after course completion, and the average number page views of their edited articles increased.

**Conclusions:**

Our results suggest that engaging pharmacy students in a Wikipedia editing assignment is a feasible alternative to writing drug monographs as a classroom assignment. Both tasks provide opportunities for students to demonstrate their skills at researching and explaining drug information but only one serves to improve wider access to quality medicines information. Wikipedia editing assignments are feasible for large groups of pharmacy students and effective in improving publicly available information on one of the most heavily accessed websites globally.

**Electronic supplementary material:**

The online version of this article (10.1186/s12909-018-1375-z) contains supplementary material, which is available to authorized users.

## Background

“Desire paths” are a concept adapted from the field of transportation planning [1]. These informal shortcuts usually represent the most easily navigated route between an origin and a destination, even if that route is not the one originally intended. Desire paths can be an amusing afterthought or an ingenious way of accommodating human nature’s propensity for self-reinforcement. For example, a number of educational institutions reportedly waited to see where students would walk regularly through the grass before deciding where to pave their campus sidewalks [2].

Just as students on these campuses demonstrate their preferred route to get to their classes, people around the world demonstrate their preferred route to get information. This route often includes accessing Wikipedia. Wikipedia is a web-based, free-content encyclopedia based on a model of openly editable content [3]. Since its launch in 2001 it has grown to be one of the most accessed websites, currently ranking 5th in the world [3, 4]. Despite the existence of more traditional and expertly validated resources, Wikipedia is used frequently by patients, students, and healthcare professionals to access medical information [5–15]. Although emerging evidence suggests some patients increasingly seek out video-based health information on sites like YouTube, [16–19] such sites remain far less frequently used than Wikipedia [14]. In this way, Wikipedia represents a “desire path” for those seeking information to improve their health or the health of the patients with whom they work.

Wikipedia contains more than 10,000 pharmacology and medicines-related articles [20]. Unfortunately, many of these have been characterized as poor quality: articles may be written at too high a readability level, include misinformation, or refer to sources that are not easily accessible because of virtual firewalls (which is permitted but less desirable); many are incomplete [21–25]. Although pundits ranging from high school teachers to the popular media have tried to deter students, patients, and healthcare professionals from using Wikipedia as a source of health information, people continue to do so [26, 27]. In response, healthcare providers and trainees have recently been encouraged to pave this particular desire path by using their technical expertise to improve the quality of Wikipedia’s health-related information [28, 29]. A compelling piece by Masukume et al discusses the value of such an approach, particularly for knowledge seekers in low- and middle-income countries [30]. Wikipedia searches are now being used with some accuracy to predict disease outbreaks [31]. Even trusted evidence networks such as Cochrane have begun to view Wikipedia as important for the dissemination of high quality information to inform health decisions [15, 32, 33].

In 2013, a group of fourth year medical students at the University of California, San Francisco (UCSF) School of Medicine took part in a novel project to edit medical Wikipedia articles for course credit. While universities had already experimented with “Wikis,” internal websites that allow users to edit content and structure, [34–36] as a means of instructing future healthcare professionals, this was the first published account of a medical school course editing directly in Wikipedia for academic credit. This short elective course resulted in the removal of poor-quality sources, the addition of better sources, the addition of new information, while allowing students to practice delivering medical information in a way that was accessible to the public [37]. Building on this model, in 2014, the UCSF School of Pharmacy investigated whether pharmacy students could improve medicines information available to a wider audience while simultaneously developing the professional skills associated with pharmacy practice. Pharmacists are well suited to editing medicines-related articles on Wikipedia [38] and US pharmacy students are required to evaluate the quality of health information as a requirement of their degree [39]. Pharmacy training programs commonly ask students to develop or edit drug monographs that summarize key information about new medicines as a curricular task.

Our team saw an opportunity to adapt the traditional educational approach of drafting drug monographs by instead having students improve information about medicines posted on Wikipedia. We hypothesized that mentored student instructors could manage the task of training enrolled students in how to edit Wikipedia. We applied the Kirkpatrick Four-Level Training Evaluation Model [40] to assess learner (a) reaction to, (b) learning from, (c) behaviors toward, and (d) results related to an educational intervention that taught pharmacy students to apply their drug information skills to editing Wikipedia pages on pharmacology topics. This project was undertaken in partnership with the Wiki Education Foundation and Wiki Project Medicine Foundation, using their freely available tools designed to support using Wikipedia for formal instruction [41].

## Methods

Our methodology was adapted from two pilot implementations (involving 232 pharmacy students editing a total of 144 Wikipedia pages) which established the feasibility of the approach. In 2014, 116 pharmacy students each edited a medicines-related page that they had chosen; in 2015, students were placed in groups of 4 to edit 30 medicines-related pages selected by the instructors based on page views and need for the drug. Specifics of these pilots are described elsewhere [42, 43]. Building on these experiences, in fall 2016 we developed a prospective cohort methodology in which third year UCSF pharmacy students (*N* = 119) in a required health policy course were assigned in groups of four to edit Wikipedia pharmacology topics (*N* = 30) selected from the World Health Organization Essential Medicines List (WHO EML). These specific pages were chosen from the WHO EML because according to Wiki Project Pharmacology’s data they had high hit rates, suggesting that the topics were of interest to the wider public, but were of low quality, suggesting that the topics would benefit from improvement efforts [20]. The specific pages edited are listed in Additional file 1.

Upon starting the project, students completed publicly available preparatory readings about the principles of Wikipedia and training on the basics of editing within the Wiki Education Foundation dashboard (approximately one hour). Next, they attended a one-hour project overview lecture (delivered by the course co-director) and a one-hour demonstration laboratory (delivered by two project students/trained peers). These training sessions included information on technical terms used by Wikipedia editors. The detailed assignment description with timelines is included as Additional file 2. Key terms necessary for interpreting the results are summarized in Table 1. We surveyed students before and after these sessions to collect anonymous reactions to the training elements. In addition to measuring perceptions, the post-training survey assessed student knowledge. Respondents were asked how to conduct edits in Wikipedia, including identifying the user sandbox, how to link terms to other Wikipedia articles, how to add citations, what references were appropriate, and what sections should be included. Following the required instruction, students were offered optional online and face-to-face activities in which to develop their skills. Details about the preparatory work, assignment stages, and specific pages edited are available on our course Wikipedia page and in the Additional files 1 and 2 [44].

**Table 1:** Key terms

Term	Definition
Sandbox	a testing environment within Wikipedia that isolates experimentation with content and structure, used before modifying an article
talk page	administrative page where editors can discuss improvements to the linked article (also: discussion page)
revision history page	webpage showing the order in which changes were made to a Wikipedia article (also: page history, edit history)
sign-off	refers to the practice of signing posts made on talk pages with your username to facilitate discussion
structural completeness	score based on how well an article matches the typical structural features (e.g. lead section, references) of a mature Wikipedia article

After completing the training, students worked in groups of four to edit their assigned Wikipedia pages. During this period of the course assignment (27 October - 17 November), and for 30 days following the assignment closure, we monitored the following metrics: 1) Wikipedia talk pages, 2) revision history pages, 3) student-initiated edits, 4) information added, 5) use of signoff procedures, 6) section changes, 7) reference changes, 8) student edits reverted, 9) change in structural completeness of the pages, and 10) citation of freely accessible (open access) references, measures that have been found to reflect improved article quality [15, 37]. We used a page views tool [45] to compare page views during the 30-day period prior to and immediately following the project. At the conclusion of the project, we administered an anonymous post-assignment survey to the students to assess attitudes. Table 2 lists the data sources we used for evaluation of the intervention; all Wikipedia data are publicly available.

**Table 2:** Data sources and elements used for evaluation

Source	Element
Surveys	Pre-assignment student survey
	Post-training student survey
	Post-assignment student survey
Article revision histories	Changes to references
	Changes to article sections
	Number of edits reverted
Article talk pages	Sign-off procedures
	Addition of accessible references
Wiki Education Foundation Dashboard	Changes in structural completeness of article
	Number of characters added
Wikipedia	Page view counter

## Results

A total of 87 students (73%) completed the pre-assignment survey, 92 students (77%) completed the post-training survey, and 80 students (67%) completed the post-assignment survey. The data that were collected on the Wikipedia edits themselves (modifications to articles, contributions to talk pages, etc.) were complete, as were data indicating page views.

### Reaction to the assignment

We used the pre-assignment survey to assess how often students used Wikipedia, as well as their knowledge and attitudes. A clear majority of respondents (87%) on the pre-assignment survey reported that they had used Wikipedia in the past, and 40% reported using it frequently. Familiarity with use did not translate into experience with adding content, however; 88% of respondents had never edited Wikipedia themselves prior to the assignment.

Students were also asked about any concerns they had about the upcoming assignment. Although 53% stated that they had no concerns, 47% expressed a range of concerns reflecting their interests and abilities. The top three areas where students expressed concern related to skills: 10% were concerned that they would engage in accidental plagiarism, 9% were concerned that they would add incorrect information, and 8% were unsure that they would be technically capable of completing the assignment.

### Learning from training and experience

**Fig. 1:**
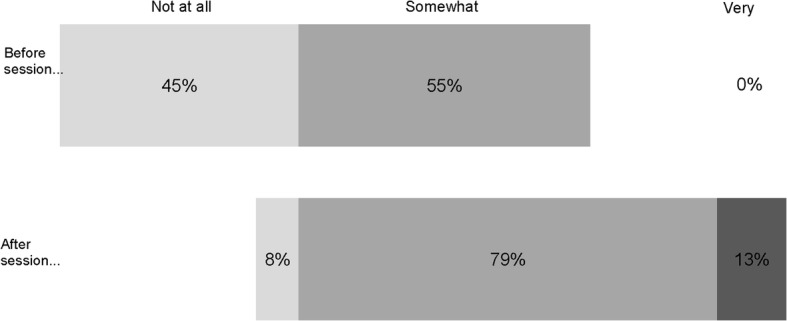
Perceived preparedness to edit Wikipedia (before and after training session)

Respondents reported that the in-class training led them to feel more confident in their approach to editing medicines-related Wikipedia pages. The pre-assignment survey asked students how prepared they felt to undertake the assignment; 45% felt “not at all” prepared. After completing the in-class training, only 8% indicated that they still felt unprepared (see Fig. 1).

**Fig. 2:**
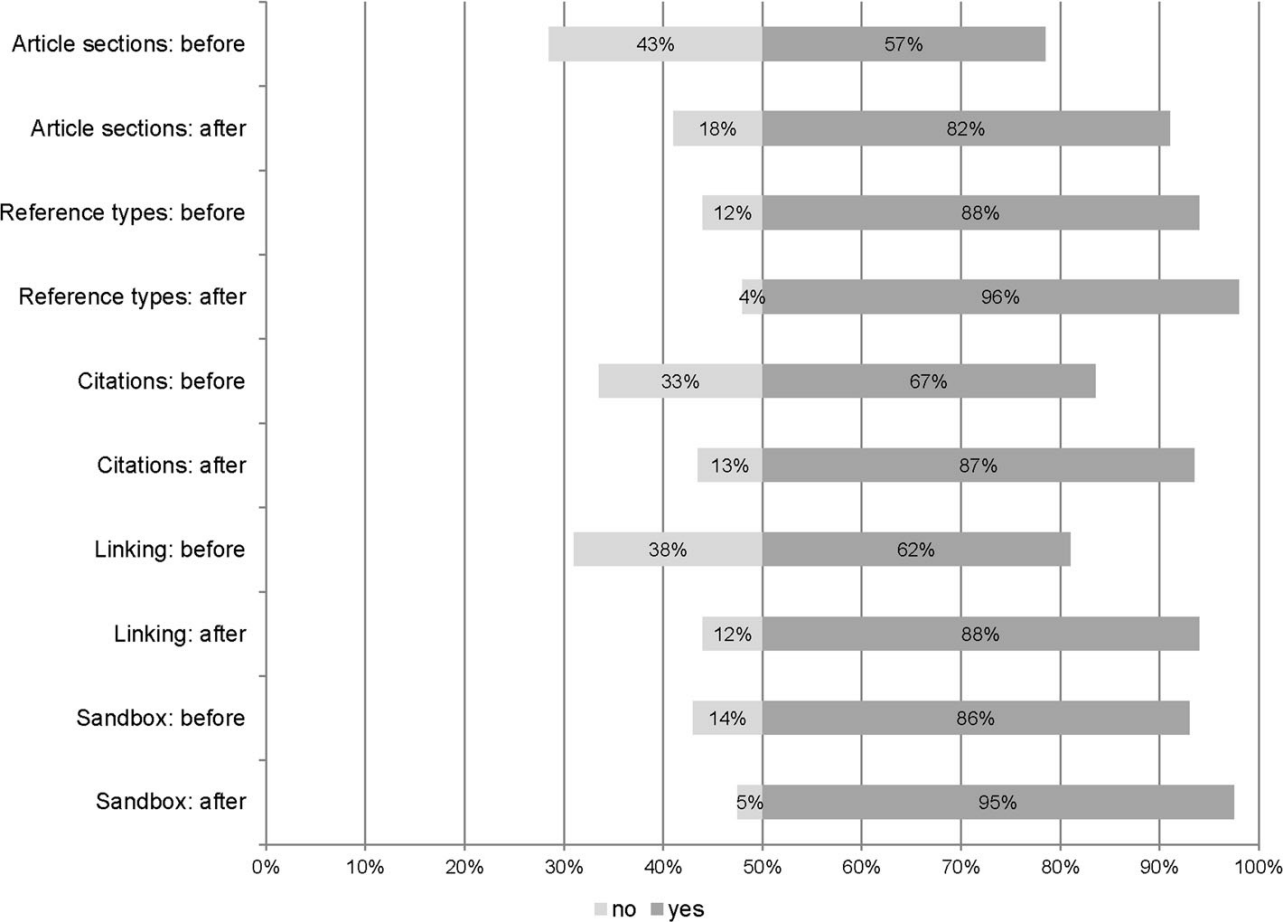
Knowledge of Wikipedia, before and after training session

The in-class training was conducted after students had reviewed the assigned Wikipedia editing videos; as a result, when students began the session, more than half understood each of these aspects. After the training, the survey assessing student knowledge found in every category—how to conduct edits, how to link terms across articles, how to add citations, what references were appropriate, how articles should be structured—more respondents stated that they could identify how to edit Wikipedia than had been able to before the assignment (see Fig. 2). Additionally, between the pre-assignment and post-training surveys, respondents demonstrated increased ability (from 52 to 63%) to identify the fundamental principles of Wikipedia. When asked whether they were required to “sign off” every talk page entry; after the training, 83% of students correctly identified that they were required to do so.

Finally, students were asked to complete a series of free-response questions, in which they provided a valid explanation in response to two prompts. When asked to describe the difference between the sandbox and drug pages, 67% of students answered the question appropriately. When asked what the user talk pages were used for, 91% of students answered appropriately.

### Actions and implementation

We assessed student aptitude with editing by identifying the changes made to sections within each article and references. All of the groups (*n* = 30) added new references, averaging 8.7 additional sources, and 80% [24] of the groups deleted an average of 4.6 existing references because they were of poor quality or there were freely accessible alternatives. When student groups worked on pages with fewer initial references, they added a greater number of references. Editing results on sections were similar; all of the groups improved some existing sections within their articles, with an average of 4.5 (range of 1–11) existing sections being improved within each article. Additionally, 80% [24] of groups added an average of 4.9 new sections.

We also evaluated student ability to follow Wikipedia’s editing practices by reviewing appropriate sign-offs on the talk pages, use of appropriate sources, and reversions of edits. Of the 30 drug pages edited, 70% had talk pages with incorrectly completed comments; an average of 1.6 comments had not been signed off appropriately. With respect to appropriate sources, 47% of the groups used only freely accessible references (suggested in the training); 53% included some sources that were not open access, with an average of 2.9 suboptimal sources per page. These suboptimal sources were identified by other groups that served as peer reviewers. Despite these incomplete sign-offs and the use of some suboptimal references, the student edits remained in place; 100% of the 30 groups had no major reverts to their edits one month after the completion of the assignment.

### Impact

Respondents on the post-assignment survey were asked to indicate how their perceptions of Wikipedia may have changed based on their participation in this activity. After editing a Wikipedia page themselves, 73% of respondents reported that they would increase their future use of Wikipedia during pharmacy school, and 48% said they viewed Wikipedia more positively than they had prior to gaining experience editing Wikipedia pages. Students whose opinions of Wikipedia changed may have been influenced by their editing experience; 9% viewed Wikipedia as less credible after learning how easy it was to change articles, 5% trusted Wikipedia more because of how well it is overseen and reviewed, and 8% said that they trusted Wikipedia only as an initial source of information. When asked about their likelihood of continuing to edit Wikipedia, 18% said they would be more likely to make edits in the future, while 58% were neutral. In the nine months following the close of the assignment, two students had actually edited further medicines pages on Wikipedia. When asked to report on what they had learned by participating in Wikipedia editing, 24% stated they had learned how to deliver information in an understandable way and 15% said that doing so was challenging. Another 15% of students reported that they learned more about finding sources that are available to the public.

The impact of student contributions on Wikipedia itself was measured by assessing the structural completeness of articles and page views. The majority of groups (70%) increased the structural completeness of their pages by an average of 32% (calculated as a proportion of the initial structural completeness). One group’s edits resulted in an 8% reduction in the structural completeness of their page, and the remaining 27% of groups contributed to their pages without increasing the degree of structural completeness. The greatest improvements were for pages with low initial levels of structural completeness. Overall, the increase in structural completeness for all groups was significant (*p* < 0.05, repeated measures t-test). The group contributions resulted in a total of 150,249 added characters to 30 Wikipedia articles, averaging 5000 characters per page. On average, page views for 29 of the 30 articles used in the assignment increased significantly after student edits, by 15% (p < 0.05, repeated measures t-test). One article (addressing pyrimethamine) was excluded from this analysis as an outlier, given that its change in page views was four times higher than the article with the next highest increase (351% v. 83%). In addition, although page views for the articles increased on average, 4 of the 30 articles saw an 8% reduction in page views after editing.

## Discussion

Wikipedia is used frequently by students, health providers, and patients for quickly accessing information even though the quality of the available information on medicines-related topics varies widely. Encouraging pharmacy students to play a role in improving these provides benefit not only to the information seekers (the users of the pages) but also to students, who gain skill in communicating information to the public. Using the Kirkpatrick Model, we evaluated the effect of mentored pharmacy students providing peer training for editing of Wikipedia medicines articles. Through questionnaires at various stages in the project, tools available within the Wikipedia pages, and via the Wiki Ed Dashboard, we found that the method of training produced positive results for both the students and the respective pages.

Students who completed the peer training had increased confidence about and improved knowledge of editing in Wikipedia. According to our results, every group was able to make a contribution by either adding new sections, improving existing sections, adding new references, or a combination of these. However, we also found that the students struggled, particularly with using the optimal types of references and when posting comments on the talk pages. The issue of identifying the best references is not unlike the challenges students experience with a traditional drug monograph assignment. Student barriers with using the talk pages, however, are unique to working within the Wikipedia platform. Our findings suggest that future preparatory training should place more emphasis on the types of references used, comparing examples of high quality, open access references with those that are lower quality or located behind virtual firewalls. Despite the fact that referencing was a challenge for some, the page contributions made by all of the groups were retained. This was an improvement on the results from the previous pilots [42, 43].

We also evaluated the effects of the assignment on the students and on Wikipedia. These results showed that the students learned more about the inner workings of Wikipedia and, as a result, many viewed this resource more critically. A small percentage of students said that they would consider editing Wikipedia pages again in the future, suggesting that their experience with the assignment was positive overall. This finding is consistent with a previous study of medical students editing Wikipedia for course credit [37]. The medical school elective, however, involved a smaller cohort (43 total students across 4 offerings of the course), a more intensive experience in which the only commitment was editing Wikipedia articles, and multiple opportunities for one-on-one and focus group feedback [37]. The nature of that course, particularly the self-selection by interested students and the intensive work with instructors, made it likely that most students would show a continuing interest in editing Wikipedia after the completion of the course. The interest in continuing to edit Wikipedia expressed by pharmacy students in a required course, which was too large to allow intensive one-on-one support, suggests that a much less intensive effort may nonetheless motivate some students to continue to improve this resource.

The impact of the student edits on the Wikipedia pages was substantial. This was supported by the improvements in the structural completeness of the pages. By improving the accuracy and comprehensiveness of the medicines pages, the public has access to better information. Importantly, since the pages also had increased page views, this amplifies the results of this intervention.

Our study has certain limitations. Although the course itself had a large number of students (*n* = 119), the assignment required students to work in groups, meaning that only 30 pages were edited. In our study, student groups were allowed to propose exactly which edits each group member contributed so it is possible that some students contributed more or better information than others. Because each group was required to summarize their proposed improvement target on the article’s Talk page in advance, each student’s edits were tracked in the Wiki Education Dashboard, and each student was assigned a distinct aspect of an assigned group’s article to peer review, the instructors were able to determine that all group members met minimum expectations even when some variability occurred.

Not all of the students engaged in the assignment attended the in-class training session and response rates for the student surveys were low, ranging from 67 to 77%. Wikipedia page views can vary seasonally and weekly (for example, they typically increase on work days) and while we made every effort to be consistent in our review, our results represent only one snapshot in time. In the case of the pyrimethamine article, given the contemporaneous media coverage, outside events may have influenced the page view impact metric [46, 47]. The assignment was integrated into a required health policy course due to fit with the existing curriculum; others may find it better aligned to different types of courses (e.g., those focused specifically on communications). We have no evidence that course type influenced the outcomes.

## Conclusions

Pharmacy schools have historically encouraged students to develop medicines information expertise by creating drug monographs. These academic exercises only serve a single purpose. As today’s students are frequent users of Wikipedia, we saw an opportunity to expand their typical experience writing drug monographs to improve medicines information on Wikipedia for the wider community. Our results suggest that this strategy is both feasible for large groups of pharmacy students and effective in improving publicly available information on one of the most heavily accessed websites globally. Pharmacy educators are encouraged to continue paving this desire path.

## Additional files

Additional file 1: Wikipedia pages edited. A list of the Wikipedia pages, medication names, and primary 479 conditions treated that were included in this project. (DOCX 19 kb)

Additional file 2: Wikipedia assignment details. A comprehensive description of the Wikipedia assignment 484 including timelines for this project. (DOCX 24 kb)

## Abbreviations

UCSF: University of California, San Francisco
WHO EML: Word Health Organization Essential Medicines List
