# Supplementary material for: Pharmacy students can improve access to quality medicines information by editing Wikipedia articles
Source: BMC Med Educ. 2018 Nov 20;18:265. doi: 10.1186/s12909-018-1375-z (PMC6245851; doi:10.1186/s12909-018-1375-z)
Supplement: Supplementary file 1 — Wikipedia pages edited. A list of the Wikipedia pages, medication names, and primary 479 conditions treated that were included in this project. (DOCX 19 kb) [file 12909_2018_1375_MOESM1_ESM.docx]

**Additional file 1: Wikipedia pages edited (2016)**

| **Wikipedia page** | **Medication name** | **Primary condition treated** |
| --- | --- | --- |
| <https://en.wikipedia.org/wiki/Abacavir> | Abacavir | HIV/AIDS |
| <https://en.wikipedia.org/wiki/Artemether> | Artemether | malaria |
| <https://en.wikipedia.org/wiki/Artesunate> | Artesunate | malaria |
| <https://en.wikipedia.org/wiki/Atazanavir> | Atazanavir | HIV/AIDS |
| <https://en.wikipedia.org/wiki/Benznidazole> | Benznidazole | Chagas disease |
| <https://en.wikipedia.org/wiki/Daclatasvir> | Daclatasvir | hepatitis C |
| <https://en.wikipedia.org/wiki/Darunavir> | Darunavir | HIV/AIDS |
| <https://en.wikipedia.org/wiki/Dasabuvir> | Dasabuvir | hepatitis C |
| <https://en.wikipedia.org/wiki/Diloxanide> | Diloxanide | amoeba infections |
| <https://en.wikipedia.org/wiki/Efavirenz> | Efavirenz | HIV/AIDS |
| <https://en.wikipedia.org/wiki/Eflornithine> | Eflornithine | African trypanosomiasis (sleeping sickness) |
| <https://en.wikipedia.org/wiki/Entecavir> | Entecavir | hepatitis B |
| <https://en.wikipedia.org/wiki/Kanamycin> | Kanamycin | tuberculosis |
| <https://en.wikipedia.org/wiki/Lamivudine/zidovudine> | Lamivudine/zidovudine | HIV/AIDS |
| <https://en.wikipedia.org/wiki/Melarsoprol> | Melarsoprol | African trypanosomiasis (sleeping sickness) |
| <https://en.wikipedia.org/wiki/Miltefosine> | Miltefosine | leishmaniasis |
| <https://en.wikipedia.org/wiki/Nevirapine> | Nevirapine | HIV/AIDS |
| <https://en.wikipedia.org/wiki/Nifurtimox> | Nifurtimox | Chagas disease |
| <https://en.wikipedia.org/wiki/Paromomycin> | Paromomycin | amebiasis |
| <https://en.wikipedia.org/wiki/Pentamidine> | Pentamidine | African trypanosomiasis (sleeping sickness) |
| <https://en.wikipedia.org/wiki/Primaquine> | Primaquine | malaria |
| <https://en.wikipedia.org/wiki/Proguanil> | Proguanil | malaria |
| <https://en.wikipedia.org/wiki/Pyrimethamine> | Pyrimethamine | toxoplasmosis |
| <https://en.wikipedia.org/wiki/Simeprevir> | Simeprevir | hepatitis C |
| <https://en.wikipedia.org/wiki/Sofosbuvir> | Sofosbuvir | hepatitis C |
| <https://en.wikipedia.org/wiki/Stavudine> | Stavudine | HIV/AIDS |
| <https://en.wikipedia.org/wiki/Suramin> | Suramin | African trypanosomiasis (sleeping sickness) |
| <https://en.wikipedia.org/wiki/Tenofovir_disoproxil> | Tenofovir disoproxil | hepatitis B |
| <https://en.wikipedia.org/wiki/Valganciclovir> | Valganciclovir | cytomegalovirus infection |
| <https://en.wikipedia.org/wiki/Zidovudine> | Zidovudine | HIV/AIDS |
