# Supplementary material for: Pharmacy students can improve access to quality medicines information by editing Wikipedia articles
Source: BMC Med Educ. 2018 Nov 20;18:265. doi: 10.1186/s12909-018-1375-z (PMC6245851; doi:10.1186/s12909-018-1375-z)
Supplement: Supplementary file 2 — Wikipedia assignment details. A comprehensive description of the Wikipedia assignment 484 including timelines for this project. (DOCX 24 kb) [file 12909_2018_1375_MOESM2_ESM.docx]

**Additional file 2: Wikipedia assignment details (2016)**

| **Action** | **Due date/time** |
| --- | --- |
| Create a personal Wikipedia account name and join the course dashboard with your Wikipedia account via link below -  https://dashboard.wikiedu.org/courses/University_of_California,_San_Francisco/Words_as_Power_-_Expanding_WikiProject_Pharmacology_(Fall_2016)?enroll=dypavjrb  * Note that your Wiki names will be associated with you personally and will be seen publicly so choose one that suggests a degree professionalism consistent with the Oath of a Pharmacist - <http://www.pharmacist.com/oath-pharmacist> | Oct 27 - 0900 |
| Select the drug your group has been pre-assigned to improve on the dashboard to link  it to your Wiki name | Oct 27 - 0900 |
| Complete the 4 required tutorials via the course dashboard:   - Wikipedia Essentials (23 mins) - Editing Basics (20 mins) - Evaluating Articles & Sources (15 mins) - Editing Medical Topics (10 mins)   Review representative medicine page with 4 paragraph lead: <https://en.wikipedia.org/wiki/Cefalexin> | Oct 27 - 0900 |
| **(Recommended)** Review the 2 brochures designed to support students in editing general and medicine-specific articles. These are useful handouts throughout the assignment.   - Editing Wikipedia - https://upload.wikimedia.org/wikipedia/commons/e/e5/Editing_Wikipedia_brochure_%28Wiki_Education_Foundation%29_%282016%29.pdf - Editing Wikipedia Articles on Medicine - <https://upload.wikimedia.org/wikipedia/commons/b/b5/Editing_Wikipedia_articles_on_medicine.pdf> | As needed |
| **(Optional)** Review 3 tutorials created during earlier implementations of this project:   - Background and General Information on Wikipedia (10 mins) - <https://vimeo.com/143296989> - Tips and Tricks for Pharmacy Students on Editing Within the Wiki Platform (8 mins) – <https://vimeo.com/143601106> - Drug Information Research Skills (11 mins) – <https://vimeo.com/143597860> | As needed |
| Participate in Wikipedia lecture/demonstration | Oct 28 - 0900 |
| **(Optional)** Participate in Wikipedia editing help session. | Nov 3 – 1300 |
| As a group, review the model page provided and then your existing page. In coordination, decide what parts you will work together to improve. Although edits are completed (and logged) individually, coordination amongst your group members will improve clarity and increase the quality of your overall edits. | Nov 3 - 1700 |
| One person from your group should insert a note in your drug’s talk page summarizing for other editors and your peer reviewers the parts of the page your group is trying to improve and specifically how you are approaching this. This can be updated as you go along. | Nov 4 - 1700 |
| Edit your page within Wikipedia. Prepare for review from your course peers and the wider Wikipedia community. | Nov 10 - 0900 |
| As a group*, perform peer review of pre-assigned drug and then individually paste/respond to the following prompts within the talk page of the drug:   1. STUDENT 1 – Does the draft submission reflect a neutral point of view? If not, specify… 2. STUDENT 2 – Are the points included verifiable with cited secondary sources that are freely accessible? If not, specify… 3. STUDENT 3 – Are the edits formatted consistent with Wikipedia’s manual of style for medicine-related articles? If not, specify… 4. STUDENT 4 – Is there any evidence of plagiarism or copyright violation? If yes, specify… 5. STUDENT 5 (if available) – Has the group achieved its overall goals for improvement?  If not, specify…   *groups should decide which student responds to each prompt | Nov 15 - 1700 |
| As a group, examine your review feedback (from course peers and the wider Wikipedia community) and use that information to finalize your submission. | Nov 17 - 0900 |
| Complete the author contributions survey (via link on learning management system).  The authorship criteria are based on those used by most academic journals - <http://www.icmje.org/recommendations/browse/roles-and-responsibilities/defining-the-role-of-authors-and-contributors.html> The key points are that authorship is based on the following 4 criteria:   - Substantial contributions to the conception or design of the work; or the acquisition, analysis, or interpretation of data for the work; AND - Drafting the work or revising it critically for important intellectual content; AND - Final approval of the version to be published; AND - Agreement to be accountable for all aspects of the work. | Nov 17 - 0900 |
